# Clinical Outcomes of an Improved Arthroscopic Suture‐Bridge Technique Assisted by a “5 O'clock” Portal for Acute Bony Bankart Lesions

**DOI:** 10.1111/os.70381

**Published:** 2026-10-01

**Authors:** Fan Jiang, Qing Yang, Chao Qi, Yi Zhang, Jinlong Ma, Tengbo Yu, Youliang Shen

**Affiliations:** ^1^ Department of Orthopedic Surgery, Qingdao Municipal Hospital Qingdao University Qingdao China; ^2^ Central Laboratories, Qingdao Municipal Hospital University of Health and Rehabilitation Sciences Qingdao China; ^3^ Department of Sports Medicine The Affiliated Hospital of Qingdao University Qingdao Shandong China; ^4^ Department of Orthopedics Affiliated Hospital of Qingdao University Qingdao China; ^5^ Traumatic Orthopedics Institute of Shandong, Affiliated Hospital of Qingdao University Qingdao China; ^6^ Department of Orthopedic Surgery, Qingdao Municipal Hospital University of Health and Rehabilitation Sciences Qingdao China

**Keywords:** arthroscopic surgery, bone healing, bony Bankart lesion, shoulder instability, shoulder joint, suture bridge technique

## Abstract

**Purpose:**

Bony Bankart lesion is a primary cause of traumatic shoulder instability. Although the arthroscopic suture bridge technique has been applied clinically, its operative complexity remains a challenge. This study aimed to evaluate the mid‐term clinical and radiological efficacy of a modified suture bridge technique combined with anchor insertion through a “5 o'clock” accessory portal for treating acute bony Bankart lesions.

**Methods:**

This study retrospectively analyzed 36 patients with acute bony Bankart lesions who underwent the modified surgical treatment between January 2019 and February 2023. All patients were followed up for at least 2 years. Preoperative and final follow‐up assessments included VAS, Constant‐Murley, ASES, UCLA, and Rowe scores, as well as shoulder range of motion (ROM). Statistical analysis was performed using paired *t*‐tests to compare preoperative and postoperative clinical scores and ROM. Fracture reduction and healing were assessed via CT 3D reconstruction at 6 months postoperatively.

**Results:**

The mean follow‐up period was 28.8 months. At the final follow‐up, all clinical functional scores and ROM showed significant improvement compared to the preoperative status (*p* < 0.001). CT 3D reconstruction at 6 months postoperatively showed successful reduction of the fracture fragment in all patients. The mean glenoid bone loss decreased from 18.2% ± 6.5% preoperatively to 2.5% ± 1.5% at follow‐up (*p* < 0.001). The fracture healing rate was 97.2%. Eighty percent of patients successfully returned to a pre‐injury or higher level of sport. The redislocation rate was 2.8%, and the total complication rate was 5.6%.

**Conclusion:**

The modified suture‐bridge technique using a “5 o'clock” accessory portal is a reliable and reproducible method for treating acute bony Bankart lesions. This approach achieves anatomical fracture reduction and yields excellent mid‐term clinical and radiological outcomes with a low risk of complications.

## Graphical abstract

This modified arthroscopic suture‐bridge technique, utilizing a unique “5 o'clock” portal, achieves reliable perpendicular fixation for bony Bankart lesions, reducing mean glenoid bone loss from 18.2% to 2.5% (*p* < 0.001) with a 97.2% healing rate and 80% return‐to‐sport rate.

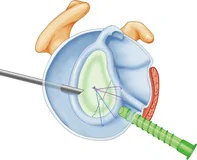

**Abbreviations**
3D: three‐dimensional
ASES: American Shoulder and Elbow Surgeons score
BFC: best‐fit circle
BMI: body mass index
Constant‐Murley: constant‐Murley score
CT: computed tomography
HAGL: humeral avulsion of glenohumeral ligaments
IGHL: inferior glenohumeral ligament
IRB: Institutional Review Board
MRI: magnetic resonance imaging
*p*
: *p* value
RF: radiofrequency
ROM: range of motion
Rowe: Rowe Rating System
SLAP: superior labrum anterior to posterior
SPSS: Statistical Package for the Social Sciences
UCLA: University of California, Los Angeles shoulder score
VAS: Visual Analog Scale

## 1 Introduction

Bony Bankart lesion is an acute shoulder injury characterized by a fracture of the anterior glenoid rim accompanying the labroligamentous complex tear, often associated with shoulder dislocation or subluxation. Acute bony Bankart lesions are typically caused by high‐energy trauma, such as sports injuries or motor vehicle accidents [1]. These lesions have been observed in up to 70% of traumatic shoulder dislocations [2].

Bony Bankart lesions directly result in anatomical and biomechanical alterations of the shoulder joint, which significantly elevate the risk of recurrent instability [3]. Studies have demonstrated that this type of injury leads to extreme instability when the glenoid bone defect exceeds 20%, highlighting that successful reduction and union of the fracture fragment are crucial for improving clinical outcomes and reducing the recurrence rate [4]. For patients with a dislocation history exceeding 1 year, fragment absorption often occurs, leading to critical glenoid bone loss and a high risk of recurrent dislocation [3, 4]. Therefore, early identification and timely treatment are paramount.

Traditional open surgical fixation is associated with several drawbacks, including larger incisions and soft tissue stripping, potential subscapularis muscle damage, limited external rotation, and post‐operative arthropathy resulting from capsular hemorrhage. These factors substantially increase the risk of stiffness and muscle weakness [2, 5]. Over the past two decades, arthroscopic surgery has been favored over open Bankart procedures due to its minimally invasive nature, particularly in cases with mild glenoid or humeral bone loss [6].

Various arthroscopic methods have emerged for treating bony Bankart lesions, including single‐row, double‐pulley, and bony Bankart bridge fixation methods [7, 8, 9, 10]. Earlier techniques primarily utilized single‐row anchor fixation, aiming to reduce and secure the bone fragment to the glenoid. Porcellini et al. presented a single‐point fixation method in which three miniRevo screws were placed along the edge of the cartilage on the healthy side of the glenoid, a cerclage‐type technique [11]. However, this single‐row fixation may not provide adequate compression, especially for larger or comminuted fragments, thereby increasing the risk of fracture non‐union or post‐operative absorption.

To enhance fixation strength, double‐row and suture bridge techniques were developed. Millett and Braun created a suture bridge construct by placing the first suture anchor on the medial side of the glenoid neck and another on the glenoid margin below the bony fragment. The suture strands were bridged across the fragment, achieving double‐point fixation and ensuring more uniform, broad‐area compression across the fracture site [12]. Similarly, Zhang et al. proposed a “double‐pulley” technique for bony Bankart lesions in 2011, which provided compression and stable fixation of the fragment by implanting anchors in the glenoid neck and under the fracture fragment, and using double mattress sutures [13]. Multiple biomechanical studies have confirmed that this double‐row fixation is superior to single‐row fixation in providing greater pullout strength and higher ultimate load‐to‐failure, significantly lowering the non‐union rate [14].

Despite the clinical efficacy of double‐row fixation and suture bridge techniques, their complexity, difficult suture management, and steep learning curve remain challenges for clinicians. Specifically, the traditional anteroinferior portal may lead to an overly oblique angle for anchor placement, making it impossible to insert the anchor perpendicular to the fracture line, which compromises the fixation strength and increases the risk of fragment displacement. Furthermore, for large fragments (> 25%), passing sutures around the fragment in the confined joint space is technically demanding, time‐consuming, and increases the risk of damage to surrounding soft tissues. These operative limitations may hinder the widespread and reproducible adoption of existing techniques in some clinical scenarios.

Herein, we present a modified arthroscopic “Bony Bankart Bridge” technique, which innovatively employs a 5 o'clock portal and a unique “medial‐lateral” fixation construct to address the aforementioned shortcomings. This modified technique is designed to ensure rigid fixation of the bone fragment through a more perpendicular anchor insertion angle and simplify the suture management process (eliminating the need for circumferential fragment lassoing), which may potentially reduce surgical difficulty and operating time based on our clinical practice. This study aims to evaluate the clinical and radiographic outcomes of this modified technique during mid‐term follow‐up and hypothesizes that it will provide reliable fracture reduction, durable clinical improvement, and achieve high patient satisfaction and return‐to‐sport rates.

## 2 Materials and Methods

### 2.1 Patient Selection

This retrospective study was approved by the Ethics Committee of the Affiliated Hospital of Qingdao University (Approval No. QYFY WZLL 42198). A total of 36 patients with acute traumatic anterior shoulder instability accompanied by a bony Bankart lesion were included. These patients underwent treatment using the “Modified Suture Bridge Technique” performed by the same senior surgeon at our department between January 2019 and February 2023. Inclusion criteria required patients to be older than 18 years, have a definitive diagnosis of traumatic anterior instability with a bony Bankart lesion classified as Bigliani Type I or Type II [15], and have a minimum postoperative follow‐up period of 2 years. Exclusion criteria included: posterior, inferior, or multidirectional instability (including habitual or chronic dislocation), glenoid bone erosion or attrition, or concomitant shoulder injuries (rotator cuff tear, SLAP lesion, or HAGL lesion). Patients with a history of prior injury or surgery on the ipsilateral shoulder, those who underwent other reconstruction procedures during the index surgery (rotator cuff repair or open fracture fixation), or those with concomitant neurovascular injury or underlying congenital, inflammatory, neurological, or degenerative shoulder diseases were also excluded. Radiological assessment using three‐dimensional (3D) computed tomography (CT) scan was performed preoperatively, immediately after the surgery, and postoperatively at follow‐up.

### 2.2 Quantification of Bone Defects

Glenoid bone loss was evaluated using CT views of the glenoid with the patient positioned supine and the affected arm in neutral rotation. After digital subtraction of the humeral head from the 3D reconstruction, the best‐fit circle (BFC) was determined using the inferior two‐thirds of the glenoid's cortical rim as the landmark. The percentage of bone loss was calculated as the ratio of the defect width to the diameter of the assumed external BFC, based on the inferior glenoid contour, and was classified as small (< 12.5% of the glenoid surface), medium (12.5%–25%), or large (> 25%) [16](Figure 1). Furthermore, the maximum depth of the potential Hill–Sachs lesion was measured on the axial CT or MRI plane as described by Saito et al. [17]. The ratio of the lesion depth to the diameter of the BFC drawn around the humeral head was utilized for analysis to minimize any variability caused by the position of the maximum depth on the humeral head or the size of the humeral head. Hill–Sachs lesions with a depth ratio of less than 10% were defined as “insignificant” [18]. Two independent observers performed the calculations to evaluate interobserver reliability for both the glenoid bone loss and the depth of the Hill‐Sachs lesions.

**FIGURE 1:**
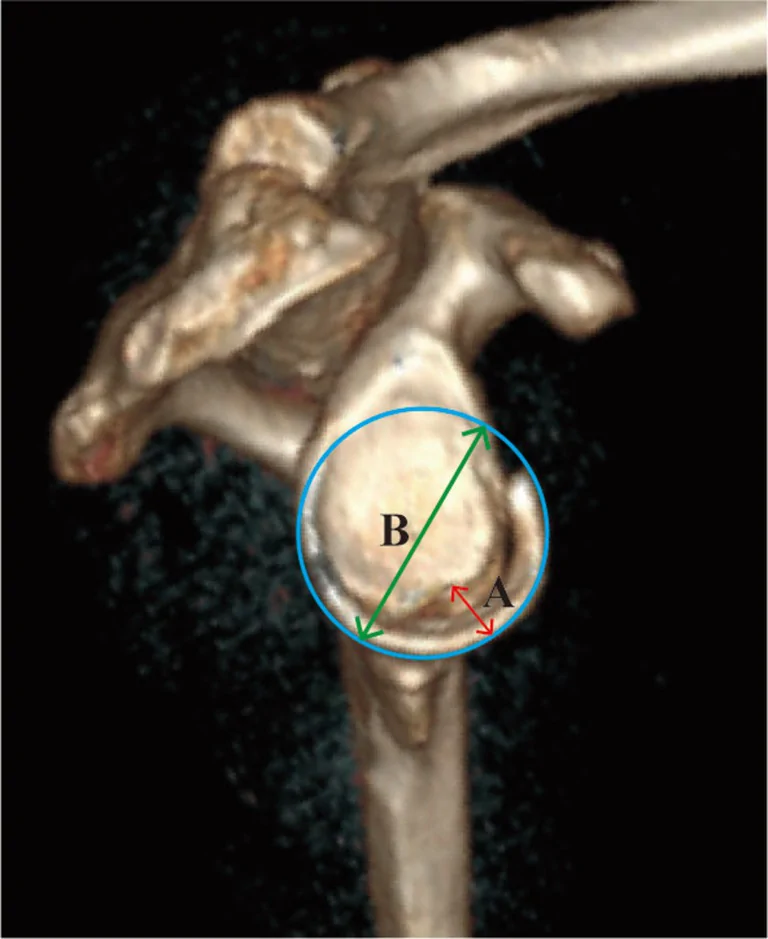
Quantification of glenoid bone loss percentage. The percentage of bone loss is calculated as *A*/*B* × 100%, where *A* represents the width of the bone defect and *B* is the diameter of the best‐fit circle of the intact glenoid.

### 2.3 Surgical Technique

All surgeries were performed by the same surgeon. The patients were placed in the beach chair position under general anesthesia. Continuous traction was applied to the affected shoulder joint using the SPIDER Shoulder Holder (Smith and Nephew) with 40° of abduction and 20° of forward flexion. A standard posterior viewing portal was established. Using the outside‐in technique, a high anterosuperior portal and an anteroinferior accessory portal were created as working portals. After arthroscopic confirmation of the bony Bankart lesion at the anteroinferior glenoid rim (3–6 o'clock region), an additional 5 o'clock anteroinferior accessory working portal was established via the outside‐in technique under direct posterior arthroscopic visualization, in accordance with the anatomical and technical specifications described by Davidson and Tibone [19]. This portal is anatomically located at the 5 o'clock position on the coronal plane of the glenoid articular face, anteroinferior to the glenoid rim and in the lower one‐third of the subscapularis muscle belly, passing slightly lateral to the conjoined tendon; its establishment maintains a safe distance from the axillary nerve (mean: 24.4 ± 5.7 mm) and musculocutaneous nerve (mean: 22.9 ± 4.9 mm) to avoid neurovascular injury. The 5 o'clock portal provides a unique, nearly vertical access angle to the anteroinferior glenoid fracture line, which is the key technical design for this modified procedure. Initially, a systematic arthroscopic examination of the joint space was performed through the posterior portal to assess the size, morphology, and position of the bone fragment, as well as the attachment of the labrum and inferior glenohumeral ligament (IGHL) complex to the fragment. These attached tissues were carefully preserved. Subsequently, a shaver and radiofrequency (RF) ablation device were used to debride the fibrous tissue from the surface of the fracture bed and the bone fragment to expose fresh bony surfaces, thereby promoting bone‐to‐bone healing. An instrument was inserted through the anteroinferior portal to elevate the bone fragment superiorly, allowing for full exposure of the anteroinferior region of the glenoid neck. Following bone fragment reduction, the first medial‐row 3.0‐mm absorbable suture anchor (Arthrex) was inserted through the 5‐o'clock working portal at a near‐perpendicular angle to the glenoid surface, positioning it at the medial margin of the bone fragment (Figure 2A). Next, the sutures of the first anchor were passed through the anterior capsule using a suture passer via the anteroinferior portal. The most inferior strand was passed through the IGHL complex as close to the 6‐o'clock position as possible to restore capsular tension. Finally, the second lateral‐row all‐suture anchor (Arthrex; PushLock) was implanted into the central “bare area” of the glenoid through the anteroinferior portal. The free ends of the medial‐row anchor's sutures were shuttled through the eyelet of the lateral‐row anchor via the anteroinferior portal, and the lateral‐row anchor was gradually tensioned (Figure 2B). As the lateral‐row anchor was tightened, the bone fragment was continuously manipulated until it was completely reduced and tightly compressed against the fracture bed, forming a double‐row suture bridge construct (Figure 2C). The separation of the bone fragment was visible arthroscopically (Figure 3A), as was the final state after fixation, where the fragment was tightly compressed against the bony bed (Figure 3B). This “one medial, one lateral” fixation pattern forms a suture bridge construct over the reduced bone fragment, with suture tension transmitted to the fragment through the attached intact capsule and labral tissue. Finally, the shoulder joint's range of motion (ROM) was tested arthroscopically to ensure no signs of impingement or instability, and the stability of the glenohumeral joint was reconfirmed.

**FIGURE 2:**
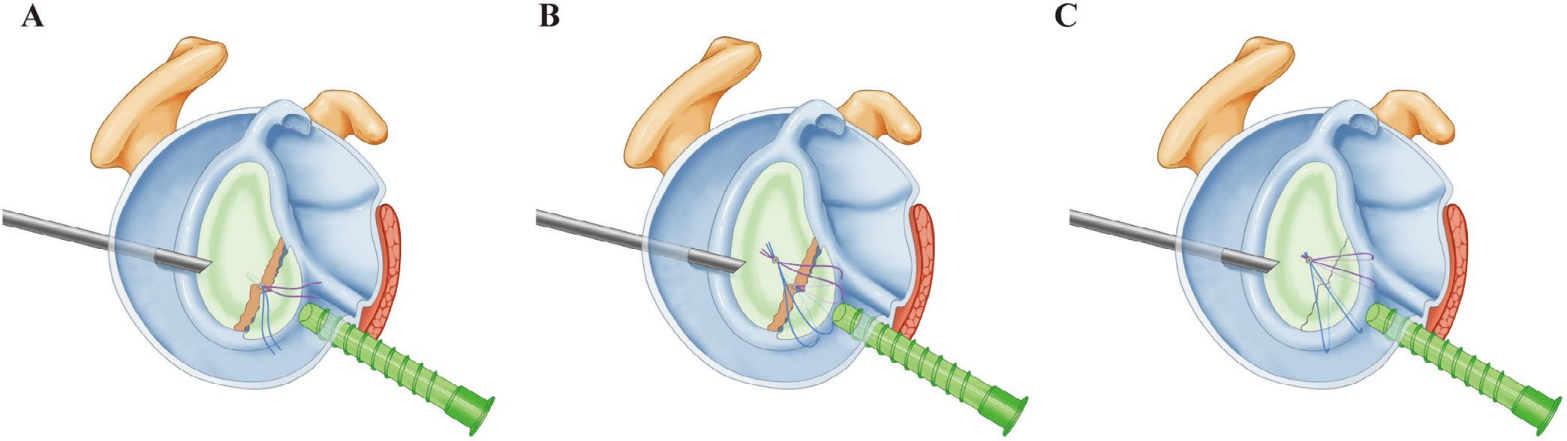
Schematic illustration of the modified bony Bankart suture bridge technique. (A) Medial‐row anchor insertion: A medial‐row absorbable suture anchor is implanted through the 5‐o'clock working portal, positioned at the inner margin of the bone fragment at a near‐perpendicular angle to the glenoid surface. (B) Suture shuttling and lateral‐row anchor insertion: After the medial‐row anchor's sutures are passed through the anterior capsule and IGHL, a lateral‐row all‐suture anchor is implanted into the central bare area of the glenoid, and the sutures are then shuttled into the eyelet of the lateral‐row anchor. (C) Final suture bridge construct: The lateral‐row anchor is tightened and compressed, forming a double‐row suture bridge construct that achieves uniform, broad‐surface compression of the fragment, resulting in secure fixation and restoration of normal anatomy.

**FIGURE 3:**
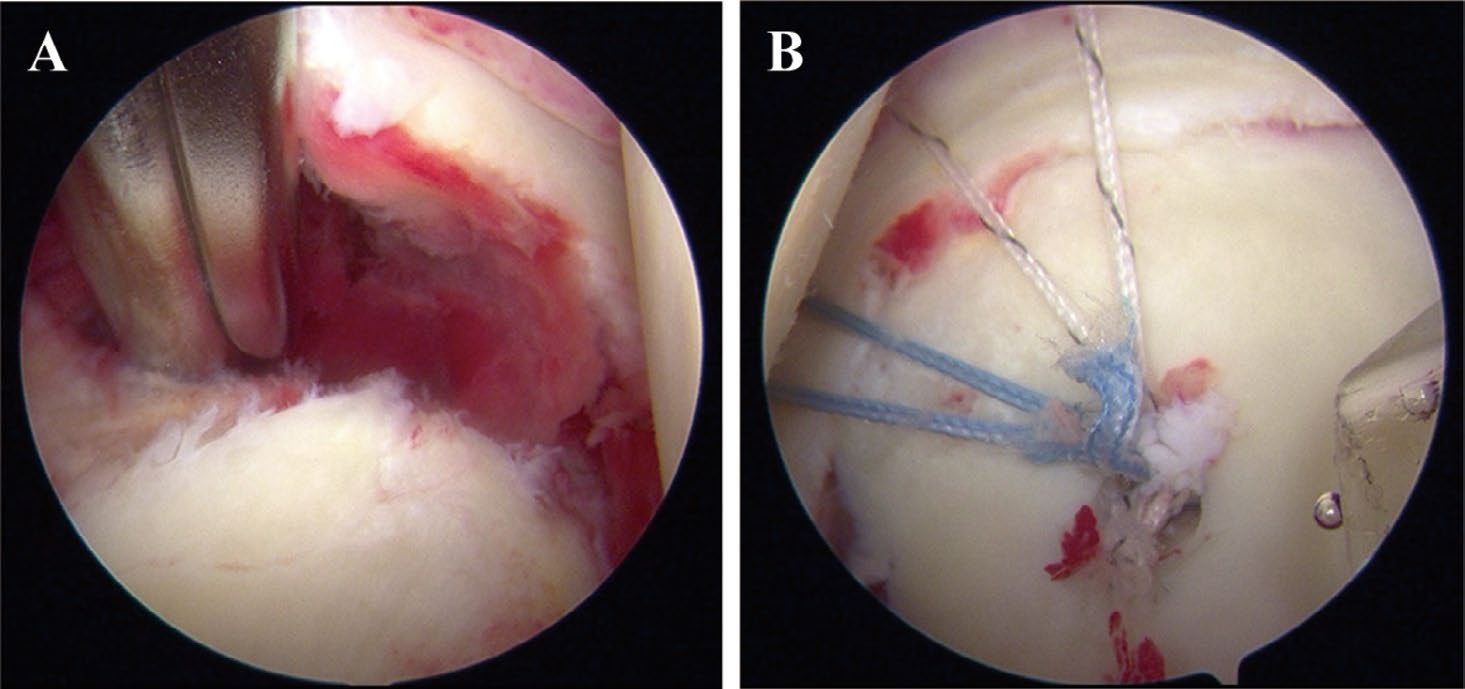
Arthroscopic repair of a bony Bankart lesion. (A) Intraoperative view showing the bony Bankart lesion and the preparation of the fracture bed. (B) Final fixation of the reduced bone fragment using suture anchors via the modified suture bridge technique.

### 2.4 Postoperative Rehabilitation

Postoperatively, the shoulders were immobilized for 3 weeks using a neutral rotation sling immobilizer to allow for the healing of the osseous injury and capsular repair. During this period, active motion of the fingers, hand, and elbow was initiated. Passive rehabilitation commenced at 3 weeks postoperatively, allowing passive motion of the shoulder joint up to 100° of forward flexion and 90° of abduction, while strictly avoiding external rotation. At 6 weeks, patients transitioned to beginning assisted active exercises, focusing on progressive forward flexion and abduction. At 3 months, a follow‐up CT scan was performed to confirm the healing and union of the fracture fragment, after which patients were permitted to return to non‐contact sports. Full return to contact or throwing sports was allowed after an average period of 6 months.

### 2.5 Clinical and Radiological Outcomes

Preoperatively, we recorded sex, body mass index (BMI), dominant arm, number of dislocation episodes, and time from injury to surgery. All patients were assessed preoperatively and at the final follow‐up to determine their ROM (forward flexion, external rotation, and internal rotation) and functional outcomes. Functional outcomes were measured using the following validated scores: the Constant‐Murley score, the Rowe Rating System (Rowe), the Visual Analog Scale (VAS) for pain, the University of California, Los Angeles (UCLA) shoulder score, and the American Shoulder and Elbow Surgeons (ASES) score. Intraoperative and postoperative complications, including but not limited to hematoma, infection, redislocation, nerve injury, or hardware failure, were recorded. The position and bony union of the bone fragment were evaluated using CT. Non‐union was defined as a visible radiolucent line < 5 mm. If the radiolucent line was ≥ 5, the bone fragment was considered to have migrated, as per established criteria [20]. Patient satisfaction was scored on a 0–10 ordinal scale, anchored at the ends of a 10‐cm line. A score of 0 represented the lowest satisfaction (dissatisfied), while a score of 10 represented the highest satisfaction. Surgical failure was defined as recurrent dislocation or subluxation of the shoulder joint that required closed reduction or revision surgery. Subjective feelings of instability or occasional clicking, without an episode of dislocation or subluxation, were not considered surgical failure.

### 2.6 Statistical Analysis

All data were statistically analyzed using SPSS (Statistical Package for the Social Sciences, version 26.0, IBM, Armonk, NY, USA). The level of significance was set at *p* < 0.05. Paired *t*‐tests were used to compare the differences between preoperative and postoperative outcome measures, including ROM, ASES score, UCLA score, and Rowe score. Furthermore, a post hoc power analysis was performed using G*Power software (version 3.1.9.7) based on the primary outcome (Rowe score). Given the significant clinical improvement observed in this study and the parameters reported in similar published series [8, 21], the calculated statistical power for our cohort of *n* = 36 was > 0.99 at an alpha level of 0.05, confirming that the sample size was sufficient to support the conclusions.

## 3 Results

### 3.1 Baseline Characteristics

A total of 36 patients with acute bony Bankart lesions were included in this study. All patients underwent treatment using the modified Suture‐Bridge technique and completed at least 2 years of follow‐up. The cohort consisted of 28 males (77.8%) and 8 females (22.2%), with a mean age of 28.5 ± 5.2 years (range: 19–45 years). All injuries were related to trauma. The mean follow‐up period was 28.8 ± 3.1 months (range: 24–36 months). Regarding general demographic and injury characteristics, all patients were preoperatively diagnosed with an anteroinferior glenoid fracture, accompanied by avulsion of the glenohumeral ligament complex, confirmed by CT 3D reconstruction. The right side was the predominant injured shoulder (18 cases, 50%), and the dominant arm was affected in 24 patients (66.7%). The most common mechanism of injury was sports activity (20 cases, 55.6%). After the initial trauma, only one dislocation episode occurred in 30 patients (83.3%). The baseline data and injury characteristics of all patients are summarized in Table 1.

**TABLE 1:** Baseline characteristics and injury profile of patients (*n* = 36).

Characteristic	Data
Age (years), mean ± SD	28.5 ± 5.2
Sex, *n* (%)	
Male	28 (77.8%)
Female	8 (22.2%)
Injured side, *n* (%)	
Right	20 (55.6%)
Left	16 (44.4%)
Dominant hand, *n* (%)	24 (66.7%)
Mechanism of injury, *n* (%)	
Sports	20 (55.6%)
Fall	12 (33.3%)
Other	4 (11.1%)
Number of dislocations, *n* (%)	
1	30 (83.3%)
> 1	6 (16.7%)
Time from injury to surgery (days), mean ± SD	15.1 ± 6.8
Mean follow‐up (months), mean ± SD	28.8 ± 3.1
Hill–Sachs lesion, *n* (%)	25 (69.4%)
Average glenoid bone loss, mean ± SD	18.2% ± 6.5%
Fracture fragment size, *n* (%)	
Small (< 15%)	15 (41.7%)
Medium (15%–25%)	18 (50.0%)
Large (> 25%)	3 (8.3%)

Diagnostic arthroscopic examination confirmed the presence of an anteroinferior bony Bankart lesion in all patients. According to the preoperative CT 3D reconstruction results, the mean glenoid bone loss was 18.2% ± 6.5%. The bone fragment size was classified as small (< 15% of glenoid width) in 15 cases (41.7%), medium (15%–25%) in 18 cases (50.0%), and large (> 25%) in 3 cases (8.3%). Furthermore, 25 patients (69.4%) also presented with a concomitant Hill‐Sachs lesion of varying degrees, none of which were defined as large lesions.

### 3.2 Clinical Outcomes

At the final follow‐up, all clinical functional scores demonstrated significant improvement compared to the preoperative status (*p* < 0.001) (Table 2). The VAS score significantly decreased from 3.5 ± 1.4 preoperatively to 0.6 ± 0.7 postoperatively. The Constant‐Murley score, ASES score, UCLA score, and Rowe score also significantly improved from preoperative values of 61.0 ± 6.5, 65.4 ± 6.5, 27.6 ± 5.5, and 52.8 ± 5.4, to 92.5 ± 3.2, 93.8 ± 3.3, 33.8 ± 2.6, and 93.2 ± 2.8, respectively. In terms of ROM, significant improvements were also observed postoperatively. Active forward flexion significantly increased from 145° ± 21° preoperatively to 168° ± 24° (*p* < 0.001). Active external rotation improved from 47° ± 8° to 61° ± 11° (*p* < 0.001). Active internal rotation improved from 28° ± 9° to 57° ± 9° (*p* < 0.001).

**TABLE 2:** Comparison of preoperative and final follow‐up clinical scores and range of motion.

Assessment	Preoperative	Final follow‐up	*p*
VAS score	3.5 ± 1.4	0.6 ± 0.7	< 0.001^***^
Constant‐Murley score	61.0 ± 6.5	92.5 ± 3.2	< 0.001^***^
ASES score	65.4 ± 6.5	93.8 ± 3.3	< 0.001^***^
UCLA score	27.6 ± 5.5	33.8 ± 2.6	< 0.001^***^
Rowe score	52.8 ± 5.4	93.2 ± 2.8	< 0.001^***^
Shoulder range of motion			
Active forward flexion (∘)	145 ± 21	168 ± 24	< 0.001^***^
Active external rotation (∘)	47 ± 8	61 ± 11	< 0.001
Active internal rotation (∘)	28 ± 9	57 ± 9	< 0.001

Abbreviations: ASES, rating scale of the American Shoulder and Elbow Surgeons; UCLA, University of California at Los Angeles; VAS, Visual Analog Scale.

^***^ *P < *0.001.

### 3.3 Radiological Evaluation

Postoperative 3D CT reconstruction results showed excellent reduction of the bone fragments in all patients. The average actual postoperative glenoid bone loss rate was significantly reduced to 2.5% ± 1.5%, demonstrating a significant difference compared to the preoperative defect (*p* < 0.001) (Figure 4). In terms of radiographic healing, 25 patients (69.4%) achieved complete bony union, 10 patients (27.8%) achieved partial union, and only 1 patient (2.8%) experienced nonunion. Among the patients with partial union, minimal bone resorption was observed, but the fracture line had largely disappeared, and the bone block position was satisfactory. The final clinical functional scores were significantly improved in all patients, which aligns with the favorable radiographic healing results, indicating that this technique can effectively restore the anatomical contour of the glenoid and thus provide a reliable foundation for shoulder joint stability.

**FIGURE 4:**
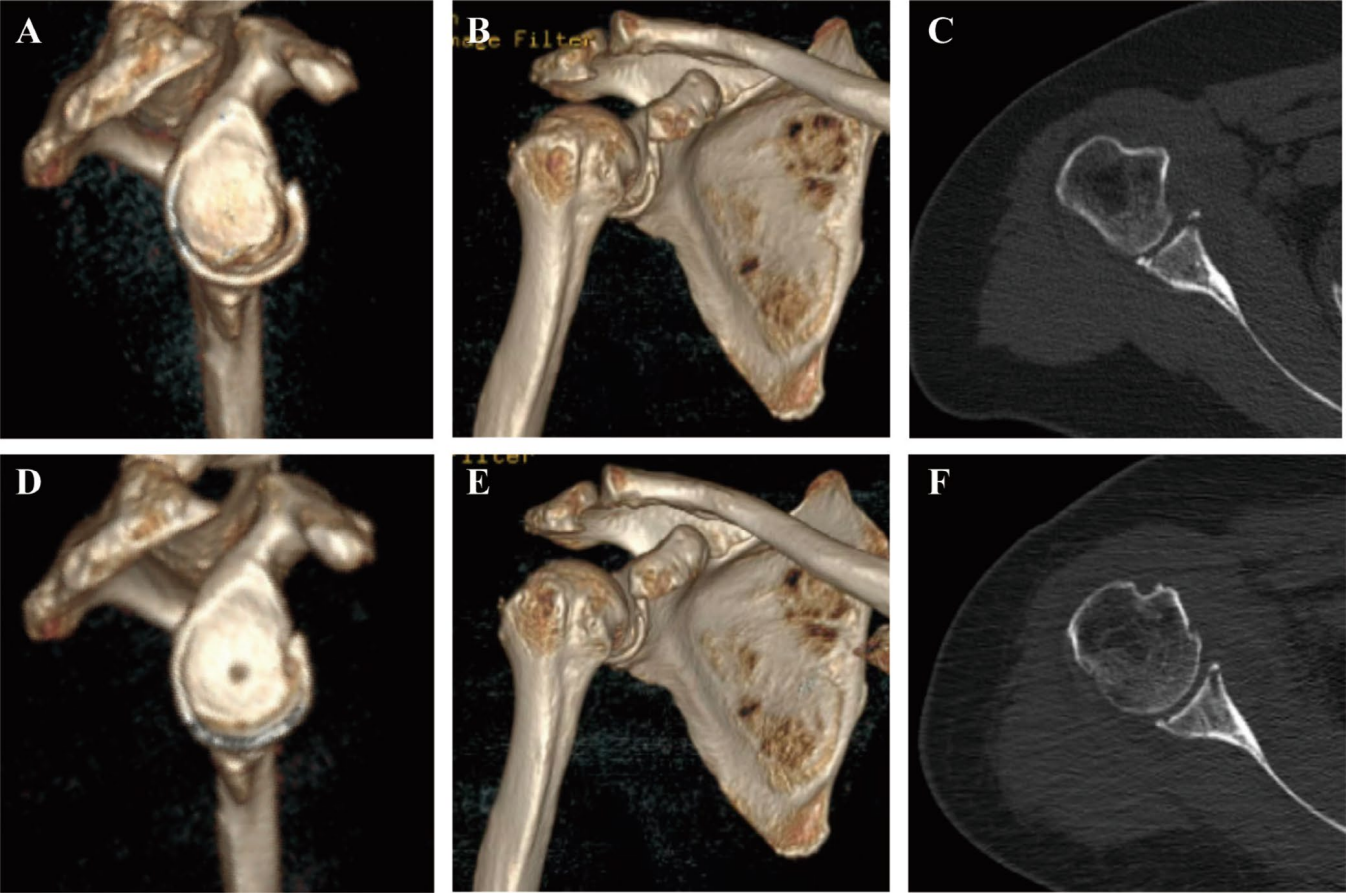
CT radiological evaluation of bony Bankart lesion. (A–C) Preoperative CT images showing the glenoid bone defect. (D–F) Postoperative CT images demonstrating successful reduction and fixation of the bone fragment. The left and middle panels display three‐dimensional (3D) reconstruction views, and the right panel displays the axial CT view.

### 3.4 Return to Sports

Among the 36 patients, a total of 20 patients (55.6%) were actively involved in sports preoperatively. At the final follow‐up, 16 patients (80%) had returned to a sports level equivalent to or higher than their preoperative level, and 3 patients (15%) returned to sports but with slight limitations. Only one patient (5%) chose not to participate in high‐intensity sports due to a fear of reinjury. Furthermore, among the patients who were actively involved in sports preoperatively, 17 patients (85%) reported that they were able to perform unrestricted overhead activities, such as throwing a baseball or basketball, without a feeling of instability. Overall, 19 patients (95%) expressed satisfaction with their postoperative shoulder function and were able to return to a comprehensive fitness program.

### 3.5 Failures and Complications

All surgeries in this study were completed smoothly, with no intraoperative complications related to the technique observed. A total of two complications (5.6%) were noted postoperatively, with no neurovascular injury, infection, or instrument‐related failures. Specifically, one patient experienced a traumatic redislocation 6 months post‐surgery due to a high‐intensity sports‐related fall. After successful closed reduction, the patient opted for conservative management and ceased high‐intensity sports participation. Another patient developed shoulder stiffness due to improper postoperative rehabilitation, but full ROM was recovered following conservative treatment and standardized physical therapy. No patients required revision surgery.

## 4 Discussion

### 4.1 Clinical Outcomes and Evolution of Fixation

This study aimed to evaluate the mid‐term clinical and radiological efficacy of a modified arthroscopic “Bony Bankart Bridge” technique in treating acute bony Bankart lesions. Our findings align with our hypothesis: the technique achieved reliable fracture reduction and significantly improved patients' clinical functional scores during mid‐term follow‐up, accompanied by a low redislocation rate and high patient satisfaction and return‐to‐sport rates. These results suggest that the modified technique is an effective and reliable treatment option for acute Bony Bankart lesions.

Bony Bankart lesions are highly susceptible to being missed on routine shoulder x‐rays, making CT 3D reconstruction crucial for assessing the location, size, classification, and guiding the treatment of the fracture fragment. The sensitivity and specificity of CT 3D reconstruction for bony Bankart lesions range from 88% to 100% [4]. Conservative treatment of bony Bankart injuries may lead to shoulder instability, with recurrence rates of up to 80%–94% [22]. Literature suggests that chronic bony Bankart fragments can undergo absorption, reaching up to 70% absorption after 2 years, leading to a significant reduction in glenoid surface area. The worse the match between the glenoid bone defect and the fracture fragment, the significantly higher the risk of dislocation [23]. Therefore, early surgical intervention is critical for preventing recurrent dislocation and maintaining shoulder stability [24].

Early arthroscopic treatment methods for bony Bankart lesions primarily involved single‐point fixation. Cameron et al. first reported the successful arthroscopic repair of a bony Bankart lesion with a 25% defect using a single suture anchor [25]. Although single‐point suture techniques have become the primary treatment for bony Bankart lesions, the literature reports inherent shortcomings and disadvantages of this method. First, single‐point fixation tends to tilt the fracture fragment towards the anchor, reducing the contact area between the fragment and the glenoid, which may impede healing. Second, placing the suture between the two bone surfaces reduces the bone‐to‐bone contact area, thus hindering fracture healing [9]. Subsequently, surgical improvements and innovations focusing on double‐point fixation techniques have continuously emerged. Millett et al. utilized a suture‐bridge technique for bony Bankart lesions, employing a two‐point fixation principle by implanting anchors in the glenoid neck medial to the fracture margin and in the articular surface lateral to it. The sutures passed through the glenohumeral ligament complex and encircled the bone fragment to achieve compression and anti‐rotation, thereby promoting fracture healing [12]. Spiegl et al. conducted a biomechanical study comparing single‐row and double‐row fixation techniques for acute bony Bankart lesions, concluding that double‐row fixation techniques improved fracture reduction and stability [26]. However, these techniques, especially traditional suture bridge techniques requiring complex suture passing and extracorporeal knot tying, still present clinical challenges such as procedural complexity, difficult suture management, and a steep learning curve. These technical limitations restrict their clinical reproducibility and surgical efficiency.

### 4.2 Technical Advantages and Surgical Pearls of the Modified Approach

The modified technique proposed in this study also achieves robust fixation of the bone fragment through the principle of two‐point fixation and double‐row anchor deployment, representing a technical variation of the traditional suture‐bridge technique with clinically observed favorable characteristics. First, it enables more precise anchor insertion: the 5 o'clock portal allows for anchor placement perpendicular to the glenoid fracture line, which ensures accurate positioning and avoids anchor slippage and insufficient holding force caused by the oblique insertion from the traditional anteroinferior portal. This advantage relies on the correct establishment of the 5 o'clock portal via an outside‐in technique under arthroscopic visualization from the posterior portal, and a cannula should be immediately placed after portal establishment to avoid potential damage to surrounding nerves during subsequent manipulation. Second, it simplifies suture management remarkably: unlike traditional techniques that demand complex suture shuttling to encircle the bone fragment, our modified “one‐inside, one‐outside” fixation pattern only requires the suture to pass through the anterior capsule of the bone fragment via a suture passer without encircling the fragment itself, which greatly reduces surgical difficulty and is particularly advantageous for injuries with bone fragments larger than 25%—a scenario where traditional suture passing is technically challenging and time‐consuming. Third, it realizes secure fixation to prevent fragment displacement: tightening the sutures applies uniform pressure to the bone fragment through the labrum and capsule, achieving anatomical reduction and stable fixation while effectively preventing suture sliding on the fragment surface and secondary displacement. To maximize this fixation effect, the lowest suture should pass through the IGHL complex as close to the 6 o'clock position as possible, which tightens the axillary pouch and restores its normal anatomical tension, a key step for maintaining glenohumeral joint stability. Fourthly, it streamlines the surgical procedure and saves operative time: the technique eliminates the need for complex extracorporeal knot tying entirely, further simplifying the surgical process, shortening operation time, and enhancing clinical reproducibility, enabling even surgeons with limited experience to master the technique after a short learning curve.

To achieve optimal outcomes with this technique, several technical pearls should be emphasized. Accurate placement of the lateral‐row anchor in the exposed cartilage‐free mid‐glenoid area is critical to prevent cleavage fractures of the anterior glenoid rim. Furthermore, the IGHL complex must be incorporated into the lowest suture—ideally near the 6 o'clock position—to restore axillary pouch tension and enhance joint stability. During the procedure, maintaining a clear field through posterior retraction of the humeral head by an assistant is essential for precise manipulation within the limited joint space.

### 4.3 Bone Healing and Cartilage Preservation

This study found that our patient cohort mainly presented with acute injuries accompanied by medium‐sized bone fragments. The double‐row bridge fixation principle of this technique provides stable, non‐tilting fixation while ensuring stable, but not overly compressed, contact between the bone fragment and the glenoid bone bed. This dynamic yet stable fixation method provides an ideal biomechanical environment for fracture healing. This may be the main reason for the high fracture healing rate, low bone absorption rate, and excellent mid‐term clinical results observed in this study. To our knowledge, this is the first study to report a modification of the double‐row bridge technique for Bony Bankart lesions, with the core innovation being the placement of the outer row anchor into the central exposed area of the anteroinferior glenoid contour. Since this area lacks cartilage coverage, our modified technique minimizes damage to the cartilage and may pose a lower risk of postoperative humeral head abrasion and related glenohumeral arthritis, with this potential benefit supported by descriptive clinical data. Furthermore, compared to single‐point fixation, our modified two‐anchor technique provides better reduction of the fracture fragment.

Notably, the risk of chondral damage induced by intra‐articular suture placement in traditional arthroscopic bony Bankart repairs. Greenstein et al. conducted a biomechanical study on cadaveric specimens and demonstrated that both single‐row and double‐row repair techniques result in measurable humeral head cartilage damage, with double‐row techniques causing significantly more severe chondral lesions (mean cartilage cutout: 57,489.1 vs. 28,763.5 μm^2^, *p* = 0.036) due to increased suture strands and direct suture‐cartilage friction [27]. This finding underscores the necessity of cartilage‐sparing surgical strategies, which is a core design principle of our modified approach. Unlike traditional double‐row techniques that require sutures to encircle the fracture fragment and lie directly over the glenoid articular surface [27], our “one‐inside, one‐outside” fixation pattern routes all sutures along the cartilage‐free bony edge and capsulolabral complex, avoiding direct contact with the weight‐bearing glenoid or humeral head cartilage. Additionally, we adopted knotless all‐suture anchors and absorbable sutures for medial‐row fixation, which aligns with the recommendation by Arce et al. that “knots and rigid anchors should be kept away from glenoid articular cartilage to prevent chondrolysis and osteoarthritis” [28]. These technical design choices fundamentally eliminate the mechanical friction between sutures and hyaline cartilage, a key mechanism of iatrogenic chondral injury identified in previous studies [27, 29]. Clinical evidence from our mid‐term follow‐up further validates the cartilage‐protective effect of the modified technique. None of the 36 patients developed clinical symptoms of glenohumeral arthritis (e.g., joint pain, crepitus) or radiological signs of cartilage erosion on 3D CT and plain x‐rays, which is consistent with the observation by Saito et al. that stable glenohumeral fixation and preservation of intact articular cartilage can facilitate glenoid remodeling and reduce degenerative risks [29]. Moreover, the lateral‐row anchor placement in the central cartilage‐free “bare area” of the glenoid avoids cartilage curettage or resection, preserving the native glenoid articular surface—an important factor for long‐term joint health, especially in young and active patients [28].

### 4.4 Study Strengths and Limitations

This study possesses several key strengths. First, it evaluates a novel and highly standardized “one‐inside, one‐outside” arthroscopic modification performed entirely by a single experienced senior surgeon, thereby eliminating inter‐surgeon technical variability. Second, the clinical findings are supported by precise, quantitative radiological assessments using postoperative 3D‐CT three‐dimensional reconstruction circle‐fitting methods to accurately document glenoid anatomical restoration and bone healing. Third, the cohort maintains a high degree of homogeneity, specifically focusing on acute lesions, which provides highly consistent mid‐term follow‐up data to reliably analyze the efficacy of this modified technique. However, this study has some limitations that should be acknowledged. First, as a retrospective single‐arm case series (Level 4 evidence), the study design focuses on describing the mid‐term outcomes of the modified technique rather than conducting direct head‐to‐head comparisons with existing methods. While we observed favorable characteristics such as simplified suture management and cartilage‐sparing effects in clinical practice, these findings are based on descriptive data and literature inference, and their relative performance compared to other arthroscopic techniques (traditional suture‐bridge, double‐pulley) remains to be verified in future comparative studies. Second, only three patients (8.3%) presented with fragments exceeding 25% of the glenoid diameter. While these patients achieved successful bony union and excellent clinical outcomes, we acknowledge that our findings may not be fully generalizable to this more challenging subgroup. In clinical practice, massive glenoid bone loss often warrants consideration for bone grafting or Latarjet procedures. However, the modified suture‐bridge technique assisted by the “5 o'clock” portal theoretically offers a biomechanical advantage for larger fragments by ensuring a more perpendicular anchor placement and broader surface compression. Future multicenter studies focusing specifically on fragments larger than 25% are needed to further validate the efficacy of this technique for massive bone defects. Third, the mean follow‐up period of 28.8 months is relatively short, and long‐term outcomes including delayed osteoarthritis, long‐term stability, and fixation durability require extended follow‐up to evaluate. Fourth, although 3D CT was used for glenoid bone loss quantification, linear measurements may not fully reflect the true anatomical features of the shallow, pear‐shaped glenoid, which could lead to minor deviations in defect assessment. Finally, no biomechanical investigation was performed in this study; we did not simulate the fixation effect on fresh specimens or evaluate mechanical differences between this technique and traditional methods, which limits further elaboration on the biomechanical properties of the modified construct.

## 5 Conclusion

In summary, our study demonstrates that the modified arthroscopic “Bony Bankart Bridge” technique exhibits favorable clinical and radiological outcomes in the treatment of acute Bony Bankart lesions. Its unique approach and fixation pattern simplify the operation, increase the rate of fracture healing and patient satisfaction, and are associated with low complication and redislocation rates. This technique is a safe, effective, and feasible treatment option, especially suitable for early intervention.

## Author Contributions

**Fan Jiang:** conceptualization, writing – original draft, methodology, data curation, investigation. **Chao Qi:** methodology, validation. **Jinlong Ma:** visualization, project administration. **Qing Yang:** investigation, methodology. **Yi Zhang:** software, formal analysis. **Tengbo Yu:** writing – review and editing, funding acquisition. **Youliang Shen:** writing – review and editing, project administration, software, conceptualization, methodology.

## Funding

This research was supported by the National Natural Science Foundation of China (Grant No. 82472431), Shandong Provincial Natural Science Foundation (Nos. ZR2024QH590, ZR2024QH588), Shandong Provincial Key R&D Program (Science and Technology Demonstration Project) in 2021 (Grant No. 2021SFGC0502), Qingdao Science and Technology Plan Project Science and Technology—Benefiting the People Demonstration and Guidance Project in 2022 (Grant No. 22‐3‐7‐smjk‐5‐nsh).

## Ethics Statement

This retrospective study was conducted with approval from the Institutional Review Board (IRB) of the Affiliated Hospital of Qingdao University (Approval No. QYFY WZLL 42198). The studies involving humans were approved by the National Center for Health Statistics Ethics Review Board of the US CDC. The studies were conducted in accordance with the local legislation and institutional requirements. The research was carried out in compliance with the ethical principles outlined in the Declaration of Helsinki.

## Consent

The participants provided their written informed consent to participate in this study. Every author has sufficiently participated in this work to take public responsibility for its content and has agreed to this submission.

## Conflicts of Interest

The authors declare no conflicts of interest.

## Data Availability

The data that support the findings of this study are available from the corresponding author upon reasonable request.
